# Supplementary material for: Asymmetric Synthesis of Quaternary Hydantoins via a Palladium-Catalyzed Aza-Heck Cyclization
Source: J Am Chem Soc. 2025 Nov 14;147(49):44692–8. doi: 10.1021/jacs.5c16022 (PMC12703750; doi:10.1021/jacs.5c16022)
Supplement: Supplementary file 2 [file ja5c16022_si_002.zip › All NMR FID Files/26/26_ProtonNMR/1/pdata/1/email_EM05-215-2-F21-55-1H_1_1.pdf]

PROTON CDCl3 /opt/nmrdata/ emeck 17

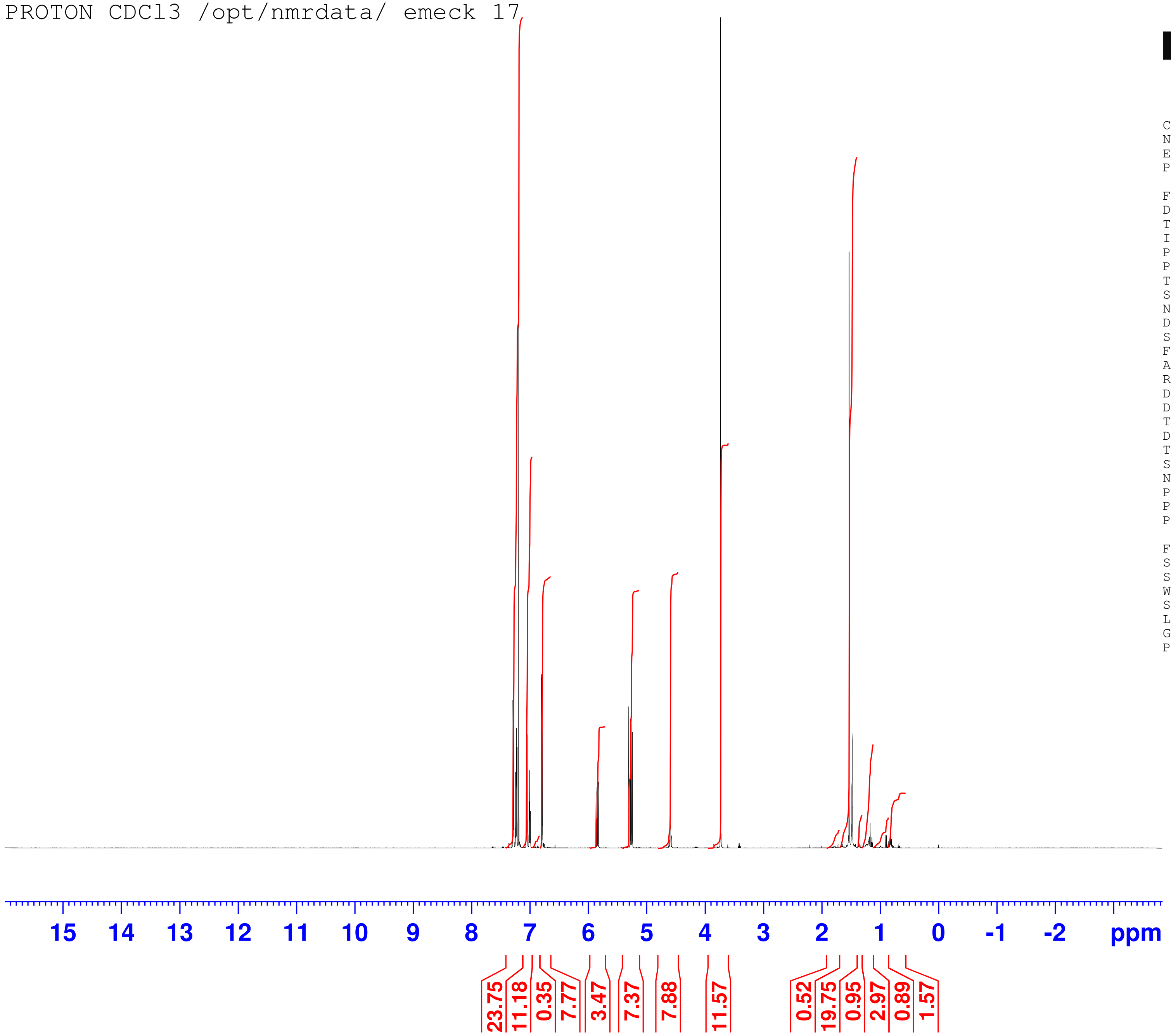

Current Data Parameters

NAME	EM05-215-2-F21-55-1H
EXPNO	1
PROCNO	1

F2 - Acquisition Parameters

Date_	20250605
Time	9.46 h
INSTRUM	Avance Neo600 U of DE
PROBHD	Z168773_0053
PULPROG	zg30
TD	65536
SOLVENT	CDCl3
NS	16
DS	2
SWH	11904.762 Hz
FIDRES	0.363304 Hz
AQ	2.7525120 sec
RG	90.5
DW	42.000 usec
DE	13.84 usec
TE	298.0 K
D1	1.00000000 sec
TD0	1
SFO1	600.3237070 MHz
NUC1	1H
P0	3.74 usec
P1	11.22 usec
PLW1	21.61000000 W

F2 - Processing parameters

SI	65536
SF	600.3200568 MHz
WDW	EM
SSB	0
LB	0.30 Hz
GB	0
PC	1.00
